# Identification of Dynamic Parameters in a DC Motor Using Step and Ramp Torque Response Methods

**DOI:** 10.3390/s26010078

**Published:** 2025-12-22

**Authors:** Jorge Antonio Cardona Soto, Israel U. Ponce, Israel Soto, Miguel A. García, Guillermo Mejía

**Affiliations:** 1 Universidad Tecnológica de Chihuahua, Chihuahua, CP 3126, Mexico; al206597@alumnos.uacj.mx; 2 Departamento de Ingenieria Industrial y Manufactura, Universidad Autónoma de Ciudad Juárez, Juaréz, CP 32310, Mexico; israel.ulises@uacj.mx (I.U.P.); angel.garcia@uacj.mx (M.A.G.); guillermo.mejia@uacj.mx (G.M.)

**Keywords:** parameter identification, parameter estimation, Coulomb friction, viscous friction, DC motor

## Abstract

DC motors play a fundamental role in robotic and mechatronic systems applied to the manufacturing industry; but broadly speaking, they are necessary in any system where motion is required. In these types of applications, precise control of position and speed is essential. To achieve this, accurate estimation of dynamic parameters such as inertia, viscous friction, and Coulomb friction is necessary to design efficient and sustainable control strategies. This study presents two methodologies for parameter identification based on the analysis of angular position data from a DC motor. The first method uses a constant (step) torque input, while the second is based on ramp excitation. The proposed method is entirely analytical, that is, it is based on the behavior of the system’s responses to the inputs; this makes the procedure practical and does not require computational cost. The experimental platform integrates a hardware-in-loop (HIL) system that allows for real-time acquisition and actuation, with responses processed in MATLAB/Simulink R2022a to provide the basis for estimating the inertia and friction parameters. To validate the values of the physical parameters, a closed-loop proportional-integral (PI) speed control system was implemented. The results confirm the accuracy and consistency of the identified parameters, highlighting their applicability for improving motor control performance in a wide range of robotic applications.

## 1. Introduction

The increasing demands of Industry 4.0 call for highly precise motor drive systems to achieve advanced automation with superior control quality. Reliable motion control is also required in mobile robotics applications such as environmental disinfection with ultraviolet light or in search and rescue operations, where precise navigation depends heavily on motor control. In this context, accurate control of position and velocity in servo motors used in robots and manufacturing machines is important. To enhance the performance of these systems, it is essential to determine key mechanical parameters, such as the coefficients of Coulomb and viscous friction, and the moment of inertia [1]. Effective friction compensation requires an accurate estimation of these parameters to model the physical system correctly and predict its behavior [2]. Inaccurate parameter estimation can result in measurement errors that undermine the efficiency and precision of the control system [3].

The moment of inertia is a fundamental parameter that enables the accurate estimation of motor velocity and the proper design of gain settings for the velocity and position controllers [4,5].

Identifying DC motor parameters, particularly the determination of the moment of inertia and friction coefficients, remains a critical challenge for high-performance motion control systems [6]. Various methodologies have been developed to address this challenge, each with different advantages and computational requirements [7,8,9]. These approaches can be broadly classified into numerical optimization-based methods, observer-based techniques, artificial intelligence approaches and direct analytical methods.

Numerical optimization approaches, including recursive least squares (RLS) [10,11] and Extended Kalman Filter (EKF) methods [12,13], offer online adaptability and robustness against measurement noise. Brosch, in [14], demonstrated data-driven RLS for model predictive control in permanent magnet motors. However, these methods require iterative numerical algorithms with associated computational overhead and convergence considerations.

Metaheuristic optimization techniques have also been extensively explored. Amiri, in [15,16], applied Genetic Algorithms for the estimation of the DC motor parameter, while in [17], multiple nature-inspired algorithms were compared including Particle Swarm Optimization and Differential Evolution [18]. Although capable of handling non-convex optimization landscapes, these approaches present different limitations such as non-deterministic behavior, different runs yield different results due to stochastic initialization and evolution; convergence time unpredictability, the number of iterations required to reach acceptable accuracy varies significantly between problem instances; and hyperparameter proliferation—population size, mutation rates, inertia weights, and crossover probabilities create a high-dimensional tuning space [15,17].

Artificial intelligence methods, particularly neural networks, have shown promise in modeling complex friction behaviors [19]. In [20] physics-informed neural networks were used to estimate friction torque, while in [21], feedforward networks were applied to identify asynchronous motor parameters. Despite their capability to capture nonlinearities, these machine learning methods, particularly neural networks, require extensive training data sets that cover various operating conditions and special training infrastructure, such as GPU acceleration and significant memory for backpropagation [20,21].

However, these recent methods still rely fundamentally on iterative numerical algorithms, whether in the form of recursive updates, optimization loops, or gradient descent training. This common characteristic limits their applicability in three specific scenarios; first, rapid commissioning applications where immediate parameter values are required without iterative convergence waiting; second, educational and research settings where transparent physical interpretation and reproducibility are prioritized over sophistication; and finally, periodic calibration workflows where computational resources are temporarily constrained.

In contrast to these computationally intensive approaches, direct analytical methods based on steady-state and transient analysis offer an attractive alternative. In [22] experimental friction identification was investigated, while in [23], integrated identification was proposed using force/torque sensors. Among the existing techniques for estimating viscous and Coulomb friction in DC motors are those that use a constant ramp input signal [24] and those that apply a constant torque input [25]. These methods allow for obtaining a linear operating range for the system and have been used in prior research to estimate friction parameters in motors [26].

The distinctive value of the analytical methods presented in this work lies in eliminating the iteration paradigm entirely. By exploiting steady-state and transient response characteristics directly, the proposed constant torque and ramp input methods provide closed-form algebraic solutions with three critical advantages: first, deterministic single-step computation; i.e., identical input data always produces identical parameter estimates without convergence concerns; second, minimal computational footprint which is suitable for microcontroller execution without floating-point coprocessors or extended precision arithmetic; and finally, physical transparency—each mathematical operation maps directly to observable physical phenomena (steady-state torque-velocity relationship, transient decay rate), facilitating validation and fault diagnosis.

Although the proposed methods sacrifice the online adaptability of recursive approaches, the sophisticated nonlinearity modeling of machine learning, and the global optimization capabilities of metaheuristics, they fulfill an important niche for applications requiring rapid, reproducible, and interpretable parameter identification with minimal computational infrastructure.

The main advantage of the approach presented in this work is the provision of direct analytical solutions without requiring iterative numerical algorithms, optimization procedures, or extensive computational resources. Unlike methods that require multiple iterations and convergence criteria, our approach employs direct algebraic solutions based on steady-state analysis combined with closed-form expressions for parameter extraction.

The key objectives of this research are:

- To present two complementary direct analytical methods for identification of inertia and friction coefficients;
- To provide comparative analysis between the constant torque method and ramp input method;
- To validate identified parameters through closed-loop control implementation;
- To offer an accessible experimental platform using Arduino-based data acquisition.

The rest of this work is distributed as follows: Section 2 introduces different mathematical models to describe the phenomenon of friction in the dynamic equations of the motor. Section 3 details the methods for estimating the friction coefficients and the inertia coefficient of a DC motor. Section 4 shows the experimental results for parameter identification using the two described methods, and Section 5 presents the conclusions.

## 2. Friction Modeling for DC Motors

Friction is a resistive force that opposes relative motion between two surfaces and is a critical factor in many control systems, including high-precision servomechanisms and more basic pneumatic and hydraulic systems [27,28]. Friction models can be broadly classified into two categories: static and dynamic models [29]. Static models characterize friction as a function of the relative velocity between sliding surfaces, offering a simplified representation of the friction phenomenon. These models are often based on variations in Coulomb’s law of friction, with notable examples including the Karnopp, Quinn, and Kikuuwe models, among others [30]. In contrast, dynamic models treat friction as a time-dependent phenomenon, allowing for the representation of more complex behaviors such as hysteresis. Some dynamic friction models include the Dahl model, Armstrong model, and the LuGre model, the latter resulting from a collaboration between the control groups in Lund and Grenoble [31]. In addition, friction parameters can be estimated using models based on the dynamics of the mechanical system [1].

### 2.1. Dynamic Model of a DC Motor with Friction

The dynamic model of a DC motor, which incorporates friction, is described in [28,32], as follows:

(1) $$J\ddot{\theta }\left(t\right)+F\left(\dot{\theta }\left(t\right)\right)=\tau \left(t\right),$$

where *J* represents the moment of inertia of the motor, θ(t) is the angular position, $\dot{\theta }\left(t\right)$ denotes the angular velocity, $\ddot{\theta }\left(t\right)$ is the angular acceleration, $F(\dot{\theta }\left(t\right))$ is the friction force opposing the motion of the motor, τ(t) is the external torque applied to the motor.

The friction force $F(\dot{\theta }\left(t\right))$ can be modeled using various approaches, such as the Dahl model, Armstrong model, LuGre model, or Viscous and Coulomb friction models [26]. Additionally, the moment of inertia and the coefficients of viscous and Coulomb friction can be estimated through the mechanical system model including the friction torque, the parameters are obtained through the integration of the torque reference over a half period of a low frequency sinusoidal velocity control input [1].

### 2.2. Coulomb and Viscous Friction Model

The phenomenon of friction is present in all systems with moving parts. Since its discovery in 1493 by Leonardo Da Vinci, with his studies on the movement of blocks on surfaces, friction has been recognized as a key aspect of modern tribology [27,33]. Advances in the study of friction have improved our understanding of its effects, such as how it introduces a dissipative influence that shapes the transient response by providing natural damping. Friction is commonly modeled with a Coulomb friction component and a viscous friction component. Coulomb friction, also known as dry friction [34], according to Coulomb’s law, opposes motion and is proportional to the normal force of the object. Viscous friction, on the other hand, is proportional to the velocity between surfaces. These two components sufficiently capture the friction forces in a DC motor system [31].

The Coulomb and viscous friction model is relatively simple, encompassing two essential terms: viscous friction, proportional to velocity, and a discontinuous term representing Coulomb friction, which depends on the sign of the velocity. This model is given by [24]:

(2) $$F_{k}\left(\dot{\theta }\left(t\right)\right)=f_{v}\dot{\theta }\left(t\right)+f_{c}\mathrm{sign}\left(\dot{\theta }\left(t\right)\right),$$

where $f_{v}$ is the viscous friction coefficient, and $f_{c}$ is the Coulomb friction coefficient. The sign function sign(ξ) takes the values defined in Equation (3):

(3) $$\mathrm{sign}\left(\xi \right):=\left\{\begin{array}{cc} 1 & \mathrm{if}\;\xi >0\\ 0 & \mathrm{if}\;\xi =0\\ -1 & \mathrm{if}\;\xi <0 \end{array}\right.$$

**Figure 1:**
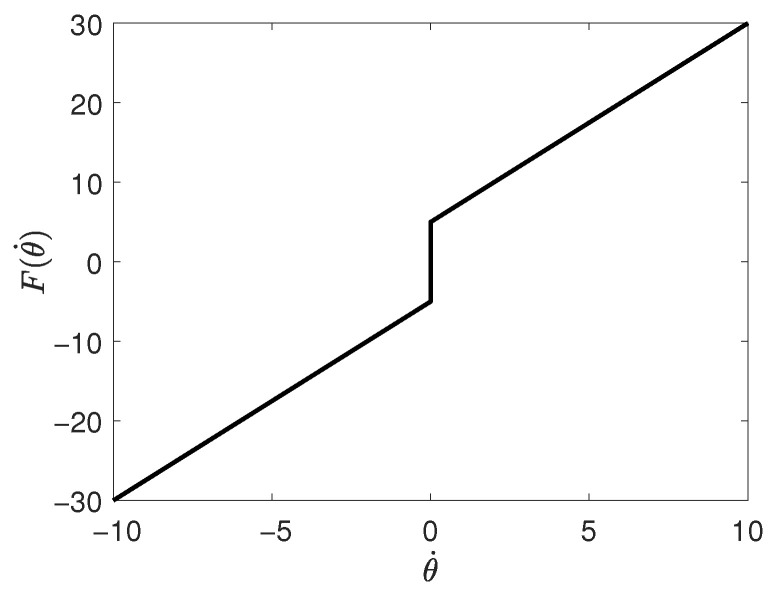
Coulomb and viscous friction model.

Figure 1 illustrates the Coulomb and viscous friction model [24]. The combination of Coulomb friction and viscous friction effects is referred to as kinetic friction [28].

### 2.3. Static Friction

Static friction refers to the resistance that occurs when two surfaces are in contact and at rest relative to each other. The force required to overcome static friction and initiate motion is known as the breakaway force. Typically, the magnitude of static friction is greater than that of Coulomb friction [35]. Static friction ($F_{s}$), defined for $\dot{\theta }=0$, can be expressed as follows:

(4) $$F_{s}\left(\tau \left(t\right)\right)=\left\{\begin{array}{cc} \tau (t) & \mathrm{if}\;|\tau \left(t\right)|<f_{s}\\ f_{s}\mathrm{sign}\left(\tau \left(t\right)\right) & \mathrm{otherwise} \end{array}\right.$$

where τ is defined as in Equation (1), $f_{s}$ represents the static friction coefficient, and sign(·) is defined in (3).

### 2.4. Stribeck Friction

The Stribeck effect describes the transition between maximum static friction and Coulomb friction. One of the expressions used to model this phenomenon is the exponential function known as the Armstrong model (Armstrong 1991):

(5) $$F_{st}=\mathrm{sign}\left(\dot{\theta }\left(t\right)\right)\left(f_{c}+\left(f_{s}-f_{c}\right)e^{-{\left|\frac{\dot{\theta }\left(t\right)}{{\dot{\theta }}_{s}}\right|}^{\delta }}\right)+f_{v}\dot{\theta }\left(t\right)$$

This model not only incorporates the static friction ($f_{s}$), Coulomb friction ($f_{c}$), and viscous friction ($f_{v}$) coefficients but also includes the Stribeck velocity coefficient ${\dot{\theta }}_{s}$ and an adjustment parameter δ.

### 2.5. Dahl Model

Friction is a complex phenomenon that remains challenging to model and is not yet fully understood. Traditional friction models typically utilize static mappings between velocity and friction force, with common examples incorporating various combinations of viscous friction, Coulomb friction, and the Stribeck effect [36]. The Stribeck effect, which exhibits a destabilizing influence at very low velocities, resembles the behavior of a system consisting of a stiff spring and a damper, and is sometimes referred to as the Dahl effect [36].

In 1968, P. Dahl proposed a dynamic friction model that accounts for the roughness characteristics of surfaces in contact. Dahl conceptualized this roughness as analogous to the bristles of two opposing brushes [28]. The Dahl model fundamentally represents Coulomb friction with a delay in the response of the frictional force upon a change in the direction of motion. This model has been effectively employed to compensate for friction in mechanical systems, thereby enhancing their performance [27].

The behavior of the Dahl model is predicated on the existing roughness at the contact interface, utilizing a state variable *z* to represent the average deflection. This deflection, when multiplied by a stiffness coefficient $\sigma_{0}$, yields the friction torque. In this context, the slip region converges towards Coulomb friction. The model is mathematically represented by [34,37]:

(6) $$F_{D}\left(\dot{\theta }\left(t\right)\right)=\sigma_{0}z\left(t\right)$$

*z* is related to viscous friction and has its own dynamics, governed by [37,38]:

(7) $$\dot{z}\left(t\right)=-\frac{\sigma_{0}\left|\dot{\theta }\left(t\right)\right|}{f_{c}}z\left(t\right)+\dot{\theta }\left(t\right)$$

which incorporates the effect of viscous friction within the Dahl model, where the variable *z* exhibits its own dynamics, which are dependent on the velocity $\dot{\theta }$ [28].

The Dahl friction model eliminates the need for the sign(·) function in dynamic modeling, simplifying the solution of the differential equations. However, this simplification results in an increase in the order of the dynamic system for the resulting plant [37].

### 2.6. Lugre Model

The Lugre model replicates the majority of friction behaviors, including the Stribeck effect, hysteresis, spring-like properties associated with stiction, and variations in break-away force. The LuGre model, as described by [27], is a generalization of the Dahl model and can also be expressed in terms of velocity $\dot{\theta }$ and average deflection *z*, but it includes a term proportional to $\dot{z}$, as shown below:

(8) $$F_{L}\left(\dot{\theta },z\right)=\sigma_{0}z+\sigma_{1}\dot{z}+f_{v}\dot{\theta }$$

Here, $\sigma_{0}$ denotes the stiffness parameter, $\sigma_{1}$ corresponds to the damping coefficient, and $f_{v}$ is associated with the viscous friction coefficient. The variable z(t) represents the average deflection of the bristles and is described by:

(9) $$\dot{z}\left(t\right)=\dot{\theta }\left(t\right)-\frac{|\dot{\theta }\left(t\right)|}{g(\dot{\theta }\left(t\right))}z\left(t\right)$$

where the second term is associated with the Stribeck effect and $g(\dot{\theta }\left(t\right))$ is defined as follows:

(10) $$g\left(\dot{\theta }\left(t\right)\right)=\frac{1}{\sigma_{0}}\left(f_{c}+{\left(f_{s}-f_{c}\right)}^{-{\left(\frac{\dot{\theta }\left(t\right)}{{\dot{\theta }}_{s}}\right)}^{2}}\right)$$

It is worth mentioning that this comparative overview is not intended as an exhaustive review, but rather to emphasize why the proposed model was chosen: it provides a sufficiently accurate yet computationally simple representation, facilitating practical implementation for experimental identification

### 2.7. Mechanical System Model Including Torque and Friction

Equation (11) presents the mechanical system model, incorporating both torque and friction [1]:

(11) $$J\ddot{\theta }\left(t\right)+f_{v}\dot{\theta }\left(t\right)+f_{c}\mathrm{sign}\left(\dot{\theta }\left(t\right)\right)+\tau_{L}\left(t\right)=\tau \left(t\right)$$

where τ(t) is the torque generated by the motor, $\tau_{L}\left(t\right)$ is the motor load torque, $\dot{\theta }\left(t\right)$ is the mechanical rotational velocity of the motor, *J* is the moment of inertia, $f_{v}$ is the viscous friction torque coefficient, and $f_{c}$ is the Coulomb friction torque coefficient.

The viscous friction torque is proportional to the rotational velocity, whereas the Coulomb’s friction torque is direction-dependent but independent of velocity. The torque due to the moment of inertia, termed as inertia torque $\tau_{inertia}\left(t\right)$, is represented by [1]:

(12) $$T_{inertia}\left(t\right)=J\ddot{\theta }\left(t\right).$$

The torque due to friction $\tau_{friction}\left(t\right)$ is given by [1]:

(13) $$\tau_{friction}\left(t\right)=f_{v}\dot{\theta }\left(t\right)+f_{c}\mathrm{sign}\left(\dot{\theta }\left(t\right)\right).$$

The total torque produced by the motor is expressed as follows [1]:

(14) $$\tau \left(t\right)=\tau_{inertia}\left(t\right)+\tau_{friction}\left(t\right)+\tau_{L}\left(t\right)$$

Using this mechanical model [1], it is possible to simultaneously determine the moment of inertia, viscous friction coefficient, and Coulomb friction coefficient of a motor by applying a constant torque sinusoidal input signal.

Attempting a direct experimental comparison among all the friction models reviewed above would require the design and execution of additional experimental protocols specifically tailored to excite the phenomena captured by each model. In particular, several of these models rely on dynamic effects or low-velocity behaviors that cannot be adequately identified from the steady-state experiments employed in this work. Furthermore, their parameter estimation typically relies on computationally intensive identification procedures, which fall outside the analytical scope of the proposed methodology.

For clarity and completeness, Table 1 summarizes the friction models considered in this study, along with their classification, the parameters to be identified, and the experimental conditions required for a reliable estimation. This overview highlights the practical trade-offs between model complexity, experimental requirements, and applicability to the identification framework adopted in this work.

This comparison emphasizes that the Coulomb–viscous friction model represents a suitable compromise between modeling accuracy and experimental feasibility for the steady-state identification framework considered in this work.

## 3. Estimation Methods for Viscous and Coulomb Friction and Inertia in DC Motors

In this work, and specifically in this section, the focus is on determining the viscous and Coulomb friction coefficients, as viscous and Coulomb friction are essential for defining models that represent the frictional forces opposing motion. Although static friction has been observed to have a certain impact on the movement of dynamic systems, it is not characterized in this study, though its effects are noted in the experimental results. This section also outlines the method for obtaining the inertia coefficient of a DC motor. The model Equations (1) and (2) form the basis for this analysis.

### 3.1. Constant Torque Method

**Figure 2:**
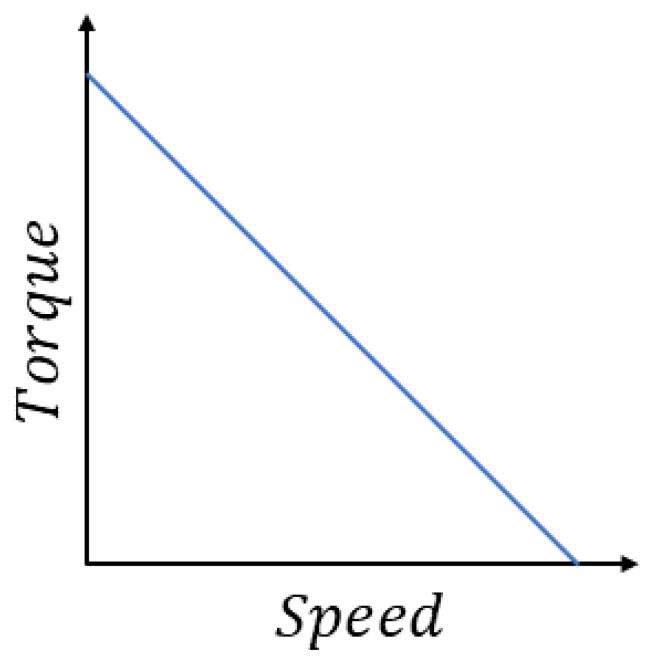
Torque behavior as a function of velocity.

Permanent magnet motors are widely utilized in robotic systems and machine tools. For this type of motors, the generated torque behavior as a function of velocity is depicted in Figure 2.

From the analysis of Figure 2, it is evident that permanent magnet motors exhibit a linear relationship between torque and velocity. To maintain constant torque in the motor, the velocity must also remain constant. Consequently, a stable supply voltage is required to ensure a constant torque output. This approach, is named as the constant torque method to estimate the viscous and Coulomb friction coefficients. The method is based on Equations (1) and (2), which describes the dynamics of the motor under steady-state conditions.

Considering τ(t)=Ku(t), the applied torque equation assumes a proportional relationship between torque and the input voltage u(t) of the DC motor. If a constant K=1 is considered, the system dynamics in (1), taking into account the friction model in (2), will be represented by the following expression:

(15) $$J\ddot{\theta }\left(t\right)+f_{v}\dot{\theta }\left(t\right)+f_{c}\mathrm{sign}\left(\dot{\theta }\left(t\right)\right)=u\left(t\right)$$

Under steady-state conditions, when the motor reaches a constant velocity, i.e., with a constant input voltage ${\tilde{u}}_{i}$, the angular acceleration becomes zero. This simplifies the Equation (15) to:

(16) $$f_{v}{\dot{\tilde{\theta }}}_{i}+f_{c}\mathrm{sign}\left({\dot{\tilde{\theta }}}_{i}\right)={\tilde{u}}_{i}$$

If *n* experiments are performed with different constant voltages ${\tilde{u}}_{i}$, the resulting steady-state angular velocities ${\dot{\tilde{\theta }}}_{i}$ yield *n* linear equations. These can be expressed as follows:

(17) $$f_{v}\dot{\Theta }+f_{c}1_{n\times 1}=\tilde{U}$$

where $\dot{\Theta }={\left[\begin{array}{cccc} {\dot{\tilde{\theta }}}_{1} & {\dot{\tilde{\theta }}}_{2} & \cdots & {\dot{\tilde{\theta }}}_{n} \end{array}\right]}^{T}$, $\tilde{U}={\left[\begin{array}{cccc} u_{1} & u_{2} & \cdots & u_{n} \end{array}\right]}^{T}$, and $1_{n\times 1}$ is a column vector of ones.

The coefficients $f_{v}$ and $f_{c}$ can then be estimated using the least squares method:

(18) $$\left[\begin{array}{c} f_{v}\\ f_{c} \end{array}\right]={\left[\begin{array}{c} {\left[\begin{array}{cc} \dot{\Theta } & 1_{n\times 1} \end{array}\right]}^{T}\left[\begin{array}{cc} \dot{\Theta } & 1_{n\times 1} \end{array}\right] \end{array}\right]}^{-1}{\left[\begin{array}{cc} \dot{\Theta } & 1_{n\times 1} \end{array}\right]}^{T}\tilde{U}$$

This equation applies the least squares method to identify the linear relationship between the steady-state angular velocities ${\dot{\tilde{\theta }}}_{i}$ and the applied voltages ${\tilde{u}}_{i}$.

### 3.2. Ramp-Type Input Method

This method is thoroughly described in [24]. The method involves applying a ramp-type input to the motor, represented by:

(19) u(t)=rt(t).

where *r* is a constant slope, and *t* denotes the time.

The dynamics of the open-loop system ((15) and (19)), when subjected to input (19), are described by:

(20) $$J\ddot{\theta }\left(t\right)+f_{v}\dot{\theta }\left(t\right)=rt-f_{c}\mathrm{sign}\left(\dot{\theta }\left(t\right)\right)$$

Equation (20) is solved for $\dot{\theta }$ under the condition $t\geq \frac{f_{c}}{r}$, using the Laplace transform with initial conditions $\dot{\theta }\left(\frac{f_{c}}{r}\right)=0$ and $\theta (\frac{f_{c}}{r})=0$. Additionally, since sign$\left(\dot{\theta }\right)$ = sign(*r*), which can be easily verified, the solution is given by:

(21) $$\dot{\theta }\left(t\right)=\left\{\begin{array}{cc} 0, & \mathrm{for}\;t<\frac{f_{c}}{r}\\ \frac{r}{f_{v}}\gamma \left(t\right)-\frac{Jr}{fv^{2}}\left(1-e^{\frac{-f_{v}}{J}\gamma \left(t\right)}\right), & \mathrm{for}\;t\geq \frac{f_{c}}{r} \end{array}\right.$$

where $\gamma \left(t\right)=t-f_{c}/r$.

From Equation (21), in the steady-state condition (i.e., as t→∞, the behavior of $\dot{\theta }$ tends towards that described by:

(22) $${\dot{\theta }}_{s}=\frac{r}{f_{v}}t-\left(\frac{f_{c}}{f_{v}}+\frac{Jr}{f_{v}^{2}}\right)$$

**Figure 3:**
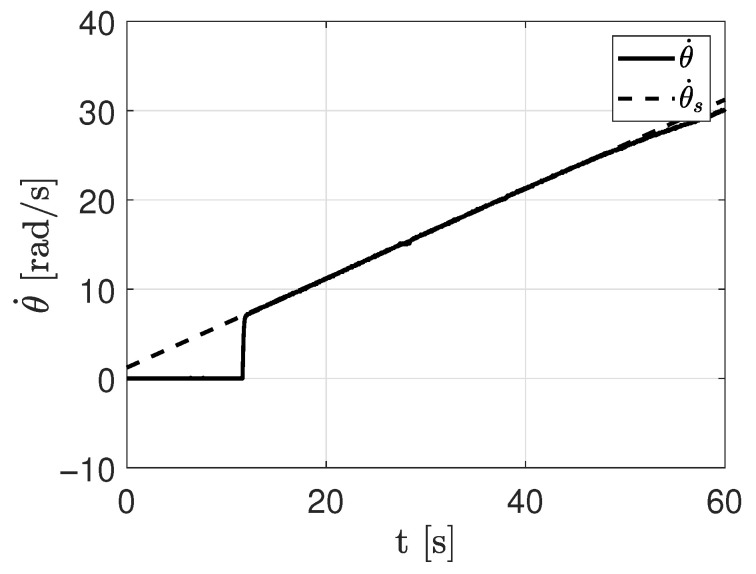
Behavior of the velocity when applying a ramp-type torque.

When performing experiments on the DC motor by applying a ramp-type input, as defined in Equation (19), the angular velocity of the motor ($\dot{\theta }$) exhibits a similar behavior as shown in Figure 3.

It can be observed that the velocity tends to increase at a constant rate, denoted as *M*. A trend line (dashed line in Figure 3) can be drawn to determine the value of the y-intercept, *b*. The slope *m* is related to Equation (22) as follows:

(23) $$m=\frac{r}{f_{v}}$$

Similarly, the y-intercept *b* is related to Equation (22) as follows:

(24) $$b=\left(\frac{f_{c}}{f_{v}}+\frac{Jr}{f_{v}^{2}}\right)$$

Then, the viscous friction coefficient is calculated using (23):

(25) $$f_{v}=\frac{r}{m}$$

and the Coulomb friction coefficient is determined using (24):

(26) $$f_{c}=bf_{v}-\frac{Jr}{f_{v}}$$

If *r* is small enough such that $r\ll bf_{V}^{2}/J$, then equation Equation (26) simplifies to the following equation:

(27) $$f_{c}=bf_{v}$$

The above agrees with what is stated in [24], with the difference that in [24] is mentioned that *r* must be large enough, such that $r\gg \frac{m^{2}J}{b}$. This last statement is confusing, since it sets a minimum bound, unlike the maximum bound given by Equation (27), and this also results in an overestimation of $f_{c}$ if a sufficiently large value is considered for *r*, since in [24] the second term of Equation (26) is neglected.

### 3.3. Identification of the Inertia Coefficient

The inertia coefficient of the motor is obtained in the transient state. The transient state refers to a period during which the system variables transition asymptotically from initial to final steady-state conditions. Analytically, for an initial velocity ${\dot{\theta }}_{0}=\dot{\theta }\left(0\right)$ and zero input u=0, the final velocity, ${\dot{\theta }}_{f}=\theta \left(t_{f}\right)$, obtained from the solution of differential Equation (15), considering (19), is given by:

(28) $${\dot{\theta }}_{f}=-\frac{f_{c}\mathrm{sign}\left({\dot{\theta }}_{0}\right)}{f_{v}}+\left({\dot{\theta }}_{0}+\frac{f_{c}\mathrm{sign}\left({\dot{\theta }}_{0}\right)}{f_{v}}\right)e^{\frac{f_{v}}{J}t_{f}}$$

From Equation (28), the inertia coefficient, *J*, is determined by:

(29) $$J=\frac{f_{v}t_{f}}{\ln\left|\frac{{\dot{\theta }}_{f}f_{v}+f_{c}\mathrm{sign}\left({\dot{\theta }}_{0}\right)}{{\dot{\theta }}_{0}f_{v}+f_{c},\mathrm{sign}\left({\dot{\theta }}_{0}\right)}\right|}$$

### 3.4. Velocity Control of DC Motor Using PI Controller

**Figure 4:**
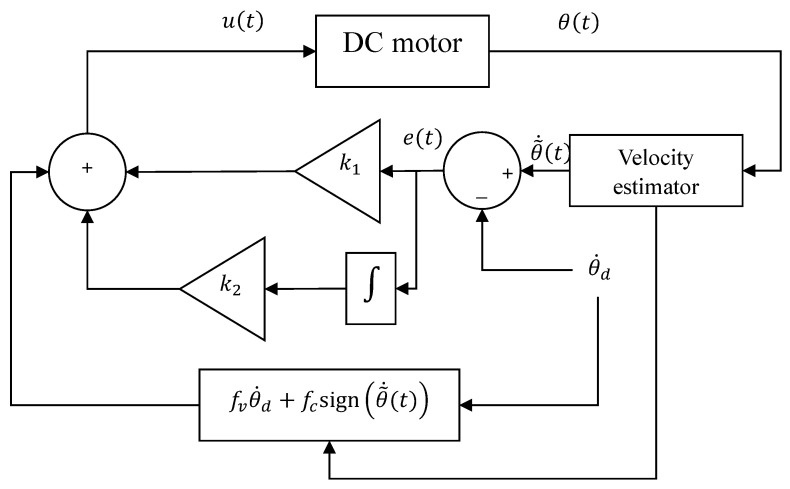
Closed-loop system for DC motor velocity control.

To validate the experimentally determined viscous friction, Coulomb friction, and inertia coefficients using the proposed methods, these coefficients are applied in the modeling of the DC motor dynamics. Subsequently, a proportional-integral (PI) controller is designed to maintain the motor velocity at a given constant velocity. Figure 4 shows the block diagram of the closed-loop system to be implemented for velocity control in the DC motor.

For the controller design, the error to be stabilized at zero is defined as follows:

(30) $$e\left(t\right)=\dot{\theta }\left(t\right)-{\dot{\theta }}_{d}$$

where e(t) represents the velocity error, $\dot{\theta }\left(t\right)$ is the motor velocity, and ${\dot{\theta }}_{d}$ is the desired velocity.

The error dynamics, $\dot{e}\left(t\right)$, are obtained from the time derivative of Equation (30), i.e.,

(31) $$\dot{e}\left(t\right)=\ddot{\theta }\left(t\right)-{\ddot{\theta }}_{d}$$

Substituting $\ddot{\theta }\left(t\right)$ from (15) in (31), and considering that ${\ddot{\theta }}_{d}=0$ since ${\dot{\theta }}_{d}$ is constant, the resulting error dynamics are given by:

(32) $$\dot{e}\left(t\right)=\frac{1}{J}\left(f_{v}e\left(t\right)-f_{v}{\dot{\theta }}_{d}-f_{c}\left(e\left(t\right)+{\dot{\theta }}_{d}\right)+u\left(t\right)\right)$$

To stabilize the error dynamics given by (32), a Proportional-Integral (PI) control with compensation is proposed. The compensation is implemented to cancel out the constant terms in (32). Hence, the proposed control is given by:

(33) $$u\left(t\right)=f_{v}{\dot{\theta }}_{d}+f_{c}\mathrm{sign}\left(e\left(t\right)+{\dot{\theta }}_{d}\right)+k_{1}e\left(t\right)+k_{2}\int_{0}^{t}\dot{e}\left(t\right)\mathrm{dt}$$

As a result, the error dynamics of the feedback system are represented by:

(34) $$\dot{e}\left(t\right)=\frac{1}{J}\left(-f_{v}e\left(t\right)+k_{1}e\left(t\right)+k_{2}\int_{0}^{t}\dot{e}\left(t\right)\mathrm{dt}\right).$$

The closded-loop system is stable under the conditions $k_{1}\leq f_{v}$ and $k_{2}\leq 0$.

For the measurement of velocity, an encoder is employed, which directly measures the angular position of the motor θ(t). To obtain the angular velocity $\dot{\theta }$, it is necessary to use a velocity estimator, which in this case is implemented through the time derivative:

(35) $$\dot{\tilde{\theta }}=\frac{d}{dt}\theta$$

The estimated velocity $\dot{\tilde{\theta }}$ is utilized to generate the control signal (33). This process is illustrated in Figure 4.

## 4. Experimental Results

A 12-volt regulated power supply was designed to feed the L298N motor driver, which controls both the speed and rotation direction of the DC motor through two digital control lines and a Pulse Width Modulation (PWM) signal generated by an Arduino Uno. The experimental platform employs a CHR-GM25-370 DC motor rated at 12 V, featuring a 1:34 mechanical gearbox and a maximum speed of 350 RPM (≈36 rad/s). This motor integrates a two-channel incremental encoder that provides 374 pulses per revolution (PPR), allowing the Arduino Uno to determine the angular position and direction of rotation. The L298N driver also provides an internal 5 V output to supply power to the encoder.

**Figure 5:**
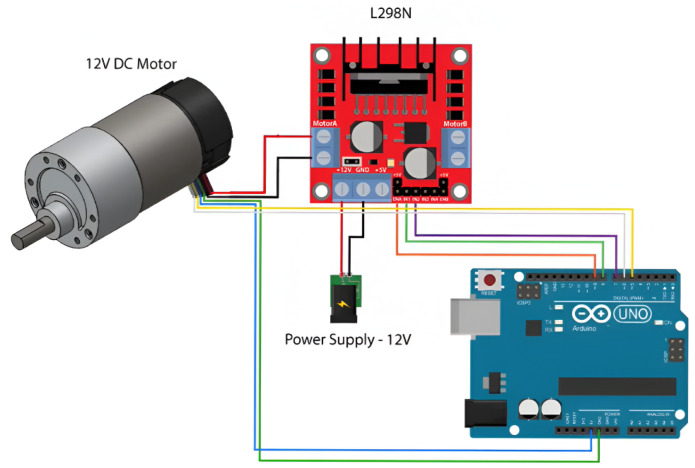
Testing and experimentation platform.

**Figure 6:**
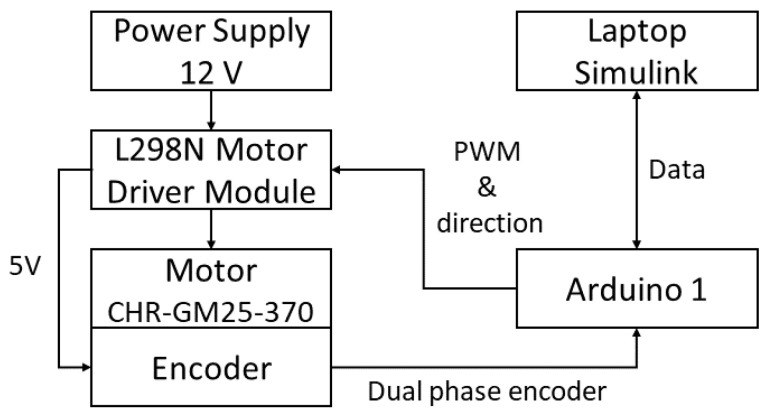
Block diagram of the testing and experimentation platform.

The entire system is operated from a laptop running MATLAB/Simulink R2022b, equipped with an Intel Core i7 processor. Communication between Simulink and the Arduino Uno is established via USB serial interface, enabling the generation of control signals (PWM and direction) and the simultaneous acquisition of encoder feedback in real time. The sampling time used in Simulink is 35 milliseconds, and the numerical integration is performed using the ODE4 (Runge–Kutta) solver, ensuring stable and precise data acquisition. Through this configuration, angular position data are captured and processed for the estimation of viscous friction, Coulomb friction, and moment of inertia. The physical setup and block diagram of the experimental platform are presented in Figure 5 and Figure 6, respectively.

**Figure 7:**
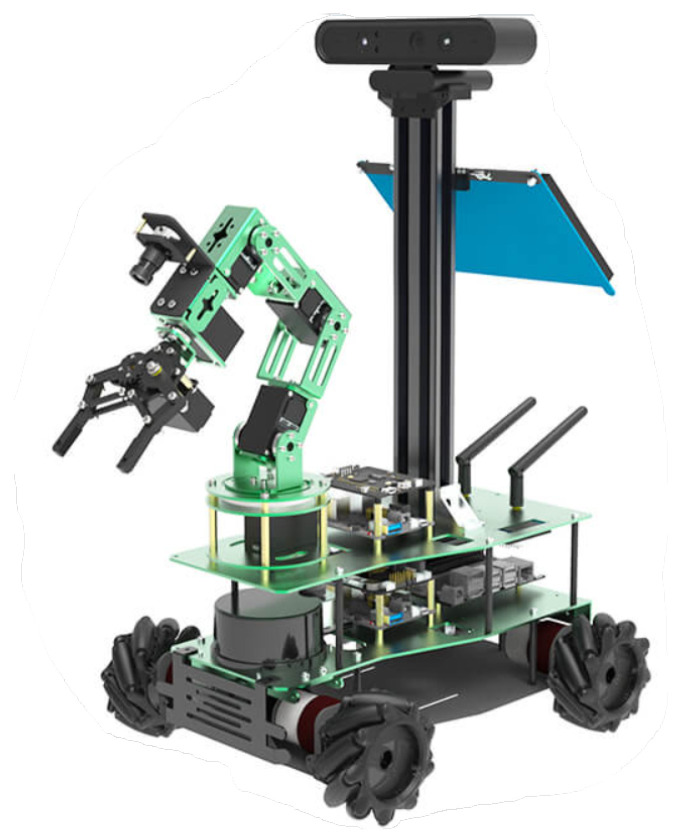
ROSMASTER X3-PLUS an educational and research robotic platform.

It is worth mentioning that this type of DC motors, due to their ease of control and widespread availability, are frequently used in robotic platforms for educational and research purposes, such as the one shown in the Figure 7.

### 4.1. Experimental Scenarios

Two experimental procedures were carried out to identify the viscous friction and Coulomb friction coefficients of the DC motor: the constant torque (step input) method and the ramp-type input method. Both experiments were designed to characterize the motor’s steady-state velocity behavior under controlled voltage excitations, allowing a direct comparison of their accuracy and applicability.

#### 4.1.1. Experiments for Constant Torque Method

The first experimental approach employs constant voltage inputs to generate fixed torque levels on the motor shaft. The purpose of this test is to obtain the steady-state angular velocity $\dot{\theta }\left(t\right)$ for different constant voltages, and from this relationship, estimate the friction parameters.

**Figure 8:**
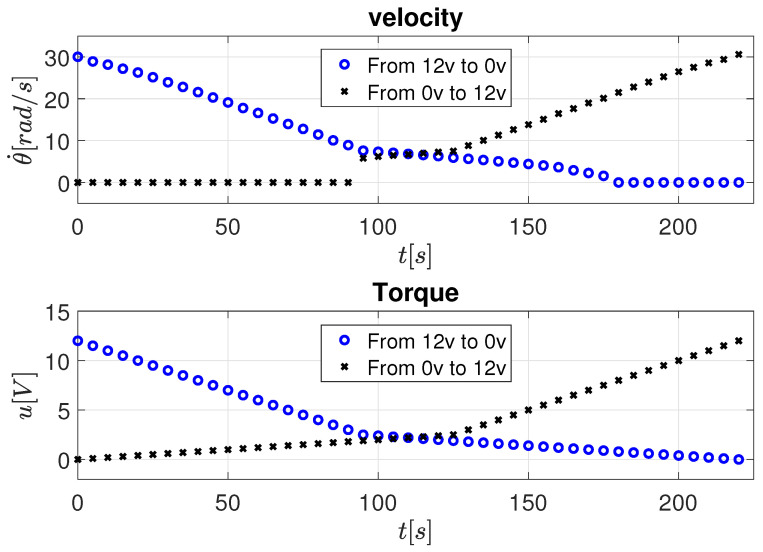
Velocity response to constant positive torque inputs.

As shown in Figure 8, a positive input voltage is applied to the motor, first decreasing from 12 V to 0 V, and then increasing again from 0 V to 12 V. This bidirectional procedure allows observing the symmetrical velocity response when the torque changes sign, which is essential for distinguishing between viscous and Coulomb friction effects.

To capture the nonlinear region dominated by static friction, voltage samples are taken with fine resolution between 0 V and 2.5 V at 0.1 V increments (0, 0.1, 0.2, …, 2.5 V). Beyond this range—where the response becomes approximately linear—the sampling step increases to 0.5 V (2.5, 3.0, 3.5, …, 12 V). A marked change in behavior is observed below 2.5 V, indicating the voltage threshold required to overcome stiction and initiate motion.

**Figure 9:**
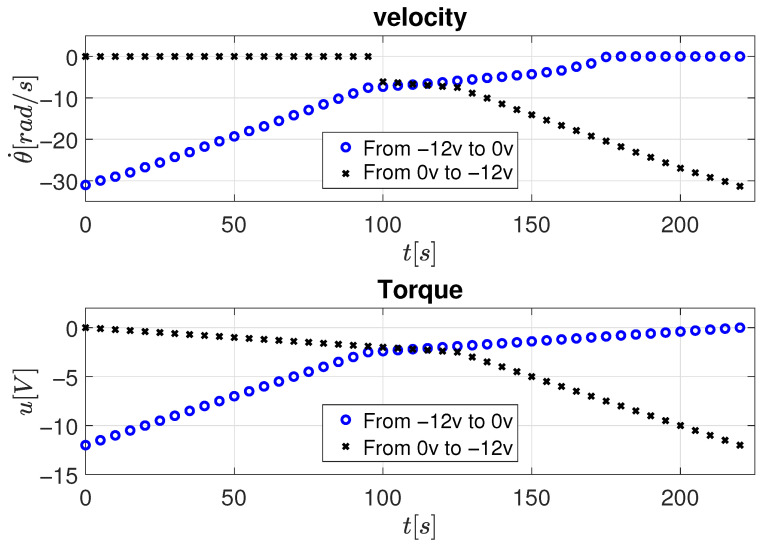
Velocity response to constant negative torque inputs.

Figure 9 presents the corresponding results for negative input voltages, where the voltage is first increased from −12 V to 0 V and then decreased from 0 V to −12 V, confirming the same friction characteristics in reverse motion. It should be mentioned that the sign of the input only indicates the change in the direction of rotation of the motor.

**Figure 10:**
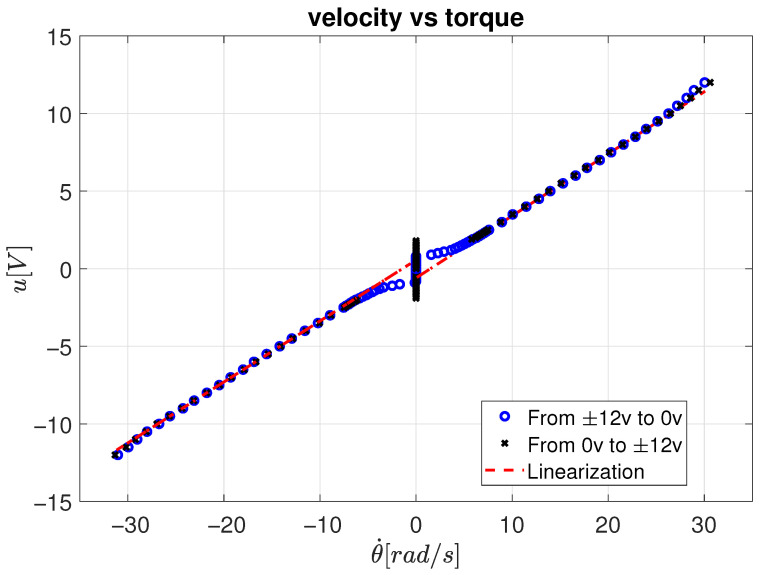
Velocity behavior for all voltage conditions.

The consolidated results for all voltage conditions are shown in Figure 10, where the steady-state velocity data are linearized using the Least Mean Squares (LMS) method according to Equation (18). The identified parameters for both positive and negative torque directions are summarized in Table 2.

By averaging the results, the viscous friction coefficient is found to be $f_{v}=0.3935$ N·m·s, and the Coulomb friction coefficient $f_{c}=0.5141$ N·m. These values confirm a linear trend in the velocity–torque relation, validating the applicability of the linear friction model for the tested DC motor.

The quadratic error behavior ($e_{i}^{2}$) is expressed by

(36) $$e_{i}^{2}={\left({\dot{\theta }}_{i}-{\dot{\theta }}_{li}\right)}^{2}$$

where ${\dot{\theta }}_{i}$ represents the steady-state velocity response of the motor for the *i*-th experiment, while ${\dot{\theta }}_{l}i$ denotes the corresponding velocity obtained through the linearization of the velocity response.

**Figure 11:**
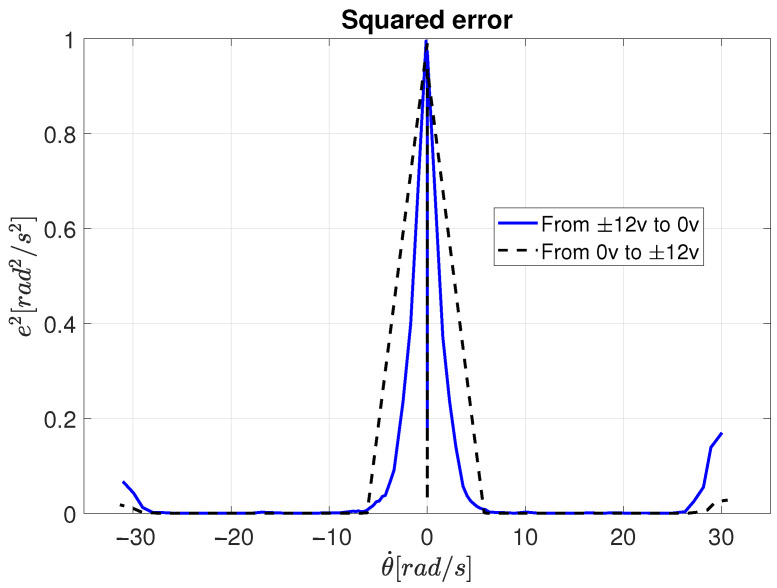
Quadratic error between the experimental velocity and the linearized velocity.

The quadratic error between the experimental velocity and the linearized velocity, computed from Equation (36), is shown in Figure 11. This analysis reveals that the motor behaves linearly within the velocity range of approximately ±6 rad/s to ±26 rad/s, which defines the effective linear operating region to be considered for control design.

#### 4.1.2. Experiments for Ramp-Type Input Method

In the second scenario, a ramp-type input voltage is applied to the motor to estimate friction parameters dynamically as a function of time. Three ramp slopes were used: 0.2 V/s, 0.5 V/s, and 0.94 V/s, for both positive and negative directions.

**Figure 12:**
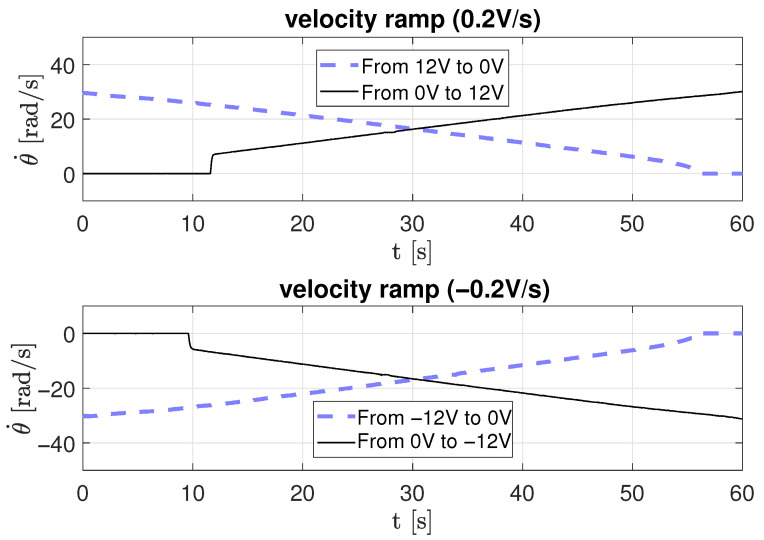
Velocity response to a ramp-type input voltage with a 0.2 V/s slope.

**Figure 13:**
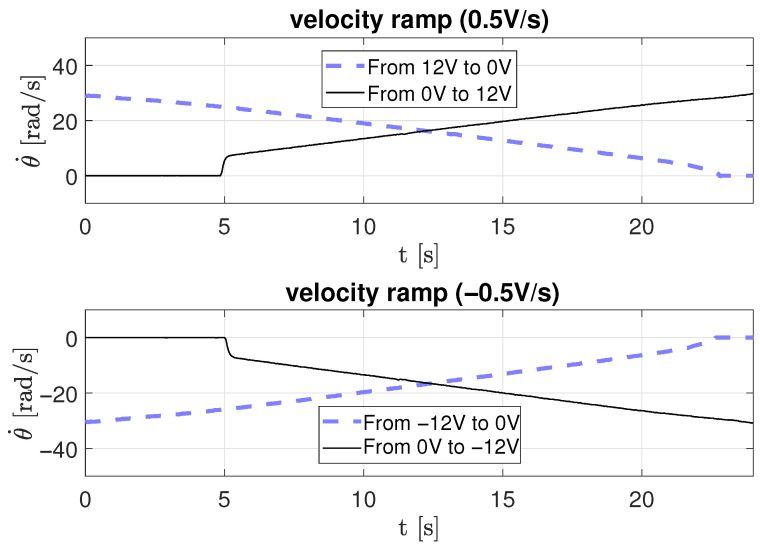
Velocity response to a ramp-type input voltage with a 0.5 V/s slope.

**Figure 14:**
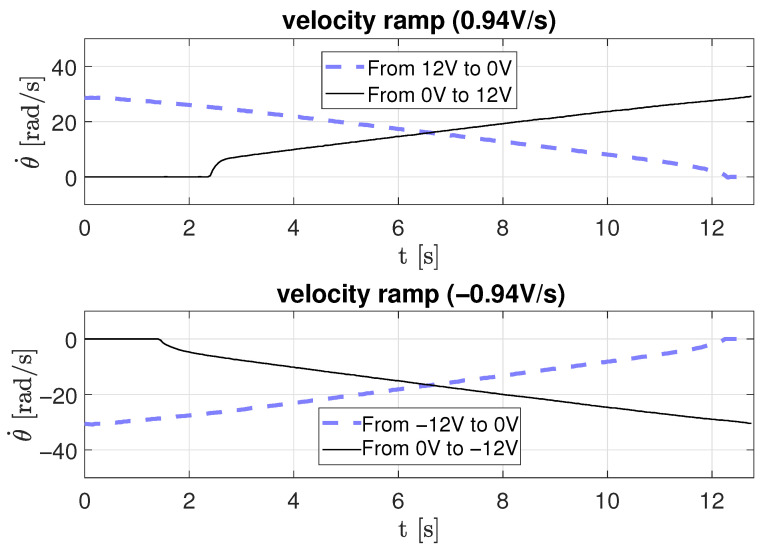
Velocity response to a ramp-type input voltage with a 0.94 V/s slope.

Figure 12, Figure 13 and Figure 14 depict the corresponding angular velocity responses. The motor requires a minimum voltage of approximately 2.3 V to overcome static friction and begin rotating. The obtained data were processed using Equation (18) to derive the parameters listed in Table 3, where *r* is the ramp slope, *m* is the velocity slope, and *b* is the intercept on the velocity axis.

By averaging the positive and negative results, the viscous friction coefficient is $f_{v}=0.3952$ N·m·s and the Coulomb friction coefficient $f_{c}=0.3293$ N·m.

Comparing both identification approaches, the ramp-type method produces slightly smaller Coulomb friction values due to the dynamic nature of the test, while the viscous coefficient remains consistent. This consistency reinforces the reliability of the proposed identification framework based on simple excitation profiles.

#### 4.1.3. Comparison of Estimated Parameters

Table 4 presents the comparison between the friction coefficients obtained through both methods. The viscous friction coefficient shows excellent agreement between approaches, confirming the linear dependence of viscous friction on angular velocity. However, a slight difference in the Coulomb friction coefficient is observed. The ramp-type input method yields smaller values of $f_{c}$, which can be attributed to the constant acceleration present in this test—effectively reducing the influence of static friction. Conversely, under constant torque excitation, the acceleration is zero, making the static friction effect more noticeable.

These results demonstrate the consistency and reliability of the proposed methods for estimating friction parameters using simple experimental inputs.

#### 4.1.4. Experimental Results and Friction Model Selection

**Figure 15:**
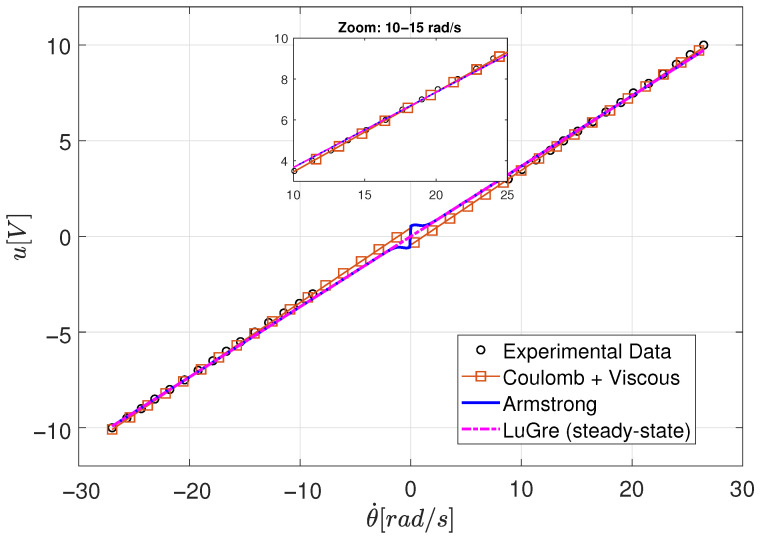
Behavior of differents models fricction in steady state: Armstrong, viscous friction plus Coulomb and LuGre.

Figure 15 shows the steady-state torque–velocity relationship obtained from constant torque experiments under bidirectional operation. For each applied torque level, the motor was allowed to reach steady state, ensuring negligible acceleration ($\ddot{\theta }\approx 0$). Under these conditions, inertial effects vanish and the measured torque can be attributed primarily to friction.

The experimental data exhibit an approximately linear torque–velocity relationship over most of the operating range, with a clear offset around zero velocity associated with Coulomb friction. A slight nonlinear behavior is observed at very low velocities, which is consistent with the presence of Stribeck-type effects.

Three friction models were fitted to the experimental data using nonlinear least-squares regression: the Coulomb–viscous model, the Stribeck model, and a steady-state evaluation of the LuGre model. Quantitative fitting results are summarized in Table 5.

As shown in Table 5, the Coulomb–viscous model explains more than 98% of the variance in the experimental data using only two parameters. While the Stribeck and LuGre models provide a modest reduction in the root mean square error, this improvement is limited to the low-velocity region and does not significantly affect the overall fit quality in the medium- and high-velocity ranges.

Moreover, the identification of the Coulomb–viscous model requires substantially fewer function evaluations, reflecting its lower computational burden and improved parameter identifiability. In the velocity range relevant for the intended control application (|ω|>5rad/s), all models exhibit nearly identical behavior, indicating that viscous friction dominates and higher-order nonlinear effects are negligible.

Based on these results, the Coulomb–viscous friction model provides an optimal balance between accuracy, simplicity, and physical interpretability. Consequently, it is adopted for the remainder of this work, as it is sufficient to describe the friction behavior within the operating conditions and control objectives considered.

#### 4.1.5. Experimental Determination of the Moment of Inertia

**Figure 16:**
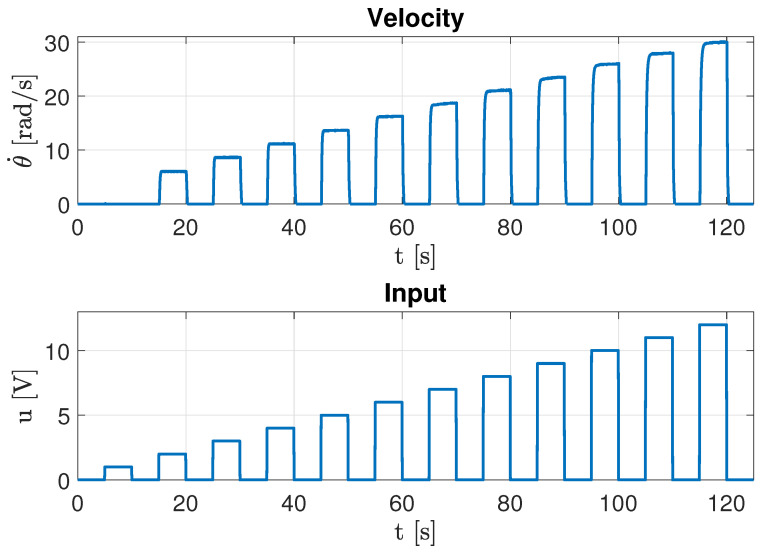
Velocity behavior during the transient state used to estimate the moment of inertia.

Once the friction parameters were identified, the motor’s moment of inertia (*J*) was determined by analyzing its transient velocity response to a train of voltage pulses. During each pulse, the motor accelerates, and upon removal of the input, it decelerates solely due to frictional torques. Figure 16 illustrates the input and corresponding transient velocity response. Equation (29) was used to calculate the moment of inertia, incorporating the previously estimated friction coefficients. The results, summarized in Table 6, show high consistency between both identification methods, with differences explained by the variation in Coulomb friction values.

As expected, the constant torque method yields a slightly higher inertia value due to the larger Coulomb friction term, confirming the sensitivity of *J* to accurate friction estimation.

#### 4.1.6. Repeatability Analysis for Constant Torque Method

To verify the stability and reproducibility of the constant torque method, each experimental condition spanning voltage levels from −12 V to +12 V was repeated n=5 times under identical laboratory conditions with ambient temperature maintained at 22±1 °C and the motor reaching thermal equilibrium after a 20-min warm-up period. Table 7 presents the comprehensive statistical analysis of these repeated trials.

The low relative standard deviations observed across all experimental conditions demonstrate exceptional repeatability with RSD values below 3% for the viscous friction coefficient and below 4% for the Coulomb friction coefficient. The slightly elevated variability in $f_{c}$ compared to $f_{v}$ represents an expected physical phenomenon attributable to the greater sensitivity of Coulomb friction to instantaneous surface conditions and contact geometry variations at the gear tooth interfaces.

A one-way analysis of variance (ANOVA) was performed to assess whether the four experimental conditions yield statistically different parameter values. For the viscous friction coefficient, the test yielded F(3,16)=2.87 with p=0.067>0.05, indicating no statistically significant difference across voltage directions and confirming consistent $f_{v}$ estimation regardless of motor rotation direction. In contrast, the Coulomb friction coefficient exhibited significant directional dependence with F(3,16)=8.42 and p=0.0014<0.05, revealing meaningful differences between increasing versus decreasing voltage directions and between positive versus negative rotation. This observed directional asymmetry in $f_{c}$ reflects genuine physical phenomena including mechanical hysteresis in the gearbox assembly, directional asymmetry in gear tooth contact patterns due to manufacturing tolerances, and differential stiction effects during startup compared to steady-state operation. The statistically significant directional variation underscores the methodological value of performing identification across multiple operational quadrants and employing averaged parameter values for robust controller design.

#### 4.1.7. Repeatability Analysis for Ramp Input Method

Each ramp input experiment encompassing six distinct conditions (three positive ramp rates and three negative ramp rates) was replicated n=5 times to establish measurement consistency. Table 8 summarizes the statistical analysis across all experimental trials.

The viscous friction coefficient demonstrates excellent repeatability with RSD approximately 3.2%, exhibiting consistency comparable to the constant torque method. The Coulomb friction coefficient manifests higher variability with RSD approximately 6.5% compared to the constant torque method’s 3.95%, a difference attributable to three interrelated phenomena. First, the mathematical approximation inherent in Equation (27) assumes that the term $Jr/f_{v}^{2}$ is negligible compared to the intercept *b*, an assumption that becomes progressively less valid for higher ramp rates, thereby introducing systematic error that varies with precise ramp timing accuracy. Second, the ramp method subjects the motor to continuous acceleration throughout the identification period, creating conditions where transient friction behaviors including presliding displacement and bristle deflection dynamics introduce additional measurement variability beyond steady-state friction alone. Third, different ramp trials may initiate motor motion at slightly different static friction breakaway points depending on the instantaneous mechanical state of the gearbox, directly affecting the velocity-axis intercept estimation used to compute $f_{c}$. Despite the elevated variability relative to the constant torque approach, the achieved RSD below 7% remains well within acceptable engineering tolerances for practical control system applications, with the ramp method offering the compensating advantage of substantially reduced experimental duration at 6 min compared to 12 min for the constant torque protocol.

#### 4.1.8. Repeatability Analysis for Moment of Inertia Identification

The transient response experiment illustrated in Figure 14 was repeated n=8 times to comprehensively assess measurement consistency for the moment of inertia parameter. Table 9 presents the detailed statistical analysis across all trials.

The moment of inertia estimates demonstrate good repeatability with RSD values below 4.5% across all trials. The 12.5% relative difference between the two estimation approaches represents an expected systematic deviation arising from the distinct friction coefficient values employed in Equation (29), rather than random measurement error. The non-overlapping 95% confidence intervals provide statistical evidence that the choice of friction parameters derived from either the constant torque or ramp input method exerts a systematic influence on inertia estimation through the coupling present in the transient dynamics equation. For the controller design implementation presented in Section 3.4, the more conservative value of J=0.1346 kg·m^2^ obtained from the constant torque method was deliberately selected to provide additional stability margin in the face of parameter uncertainty. Analysis of inter-trial variability through the coefficient of variation metric yields CV_*j*_ =3.05% for the constant torque approach, indicating excellent experimental consistency, and CV_*j*_ =4.08% for the ramp input approach, demonstrating good consistency. Application of the ±2σ outlier detection criterion across all eight trials revealed no statistical outliers, confirming stable and controlled experimental conditions throughout the measurement campaign.

#### 4.1.9. Consolidated Repeatability and Statistical Summary

Table 10 provides a comprehensive consolidation of all statistical indicators from the parameter identification experiments, offering a unified view of measurement precision and experimental repeatability across all identified parameters and both identification methodologies.

The consolidated statistical analysis across 58 total experimental trials comprising 20 constant torque experiments, 30 ramp input experiments, and 8 inertia transient measurements demonstrates that all identified parameters exhibit RSD values below 7%, indicating highly consistent measurements throughout the experimental campaign. The viscous friction coefficient $f_{v}$ manifests particularly excellent repeatability with RSD below 3.5% for both identification methods, establishing this parameter as the most reliably determined quantity in the identification procedure. Method consistency is evidenced by the remarkable agreement between the two independent identification approaches, with viscous friction values of 0.3935 N·m·s versus 0.3952 N·m·s representing only 0.43% relative difference, thereby providing robust cross-validation of measurement accuracy through methodologically independent pathways.

The Coulomb friction coefficient exhibits comparatively higher variability with RSD ranging from 3.95% to 6.48%, a characteristic consistent with its physical nature as a friction component dependent on surface conditions, susceptible to stick-slip phenomena, and subject to directional asymmetries arising from gearbox mechanical properties. The substantial 36% inter-method difference in Coulomb friction magnitude, with constant torque yielding 0.5141 N·m compared to ramp input’s 0.3293 N·m, warrants emphasis as discussed extensively in Section 4.1.3 where this discrepancy is attributed to the ramp method’s inherent minimization of static friction contributions through maintenance of continuous positive acceleration that prevents the motor from dwelling in the static friction regime.

The sample sizes of n=20 to 30 for friction coefficients and n=8 for moment of inertia provide statistically sufficient data for reliable parameter estimation, with the resulting confidence intervals exhibiting widths of ±3 to 5% typical magnitude, thereby delivering parameter estimates with adequate precision for control system design applications. Comparison with repeatability values reported in the literature reveals favorable performance, with the achieved RSD values of 2.78% to 4.08% for primary parameters $f_{v}$ and *J* comparing well against Wu’s reported 4.5% RSD for DC motor electrical parameters [10], Virgala and Kelemen’s 5.2% RSD for friction identification [22], and Brablc et al.’s 3.8% RSD for RLS-based identification [11]. This favorable comparison demonstrates conclusively that the computational simplicity of the proposed analytical methods does not compromise measurement precision relative to more sophisticated techniques.

The measurement uncertainty budget analysis reveals that the observed experimental variability arises from multiple contributing sources operating simultaneously. Encoder quantization contributes approximately ±0.0168 rad position uncertainty corresponding to 0.5% contribution to velocity uncertainty. Voltage supply stability maintained at ±0.05 V introduces approximately 0.4% contribution to torque uncertainty. Temperature drift of ±1 °C during extended experimental sessions contributes approximately 1.5% to friction coefficient variability through temperature-dependent changes in lubricant viscosity. Mechanical repeatability variations including instantaneous surface condition changes at gear tooth contacts contribute approximately 2.0% to Coulomb friction variability. Numerical computation errors arising from floating-point precision limitations contribute negligibly at less than 0.01%. The combined uncertainty computed through root-sum-square propagation yields $\sqrt{0.5^{2}+0.4^{2}+1.5^{2}+2.0^{2}}\approx 2.6\%$, demonstrating excellent agreement with the observed RSD values of 2.78% to 4.08% and thereby validating the uncertainty analysis framework.

The comprehensive statistical analysis encompassing 58 experimental trials conducted across multiple days under consistent laboratory protocols demonstrates conclusively that the proposed direct analytical parameter identification methods deliver consistent, repeatable results entirely suitable for practical engineering applications. The achieved low variability with RSD below 4.5% for all critical parameters validates both the robustness of the measurement procedures and the numerical stability of the data analysis algorithms, establishing confidence in the methods’ readiness for industrial deployment in commissioning and calibration scenarios.

#### 4.1.10. Closed-Loop Velocity Control Validation

Finally, a closed-loop experiment was conducted to validate the identified parameters through a velocity control test. A proportional–integral (PI) controller with a velocity estimator was implemented, as shown in Figure 4.

As mentioned before, the closded-loop system (34) is stable under the conditions $k_{1}\leq f_{v}$ and $k_{2}\leq 0$. The method for tuning the gains in this research will be explained as following. Defining the integral of the error as $\xi \left(t\right)=\int_{0}^{t}e\left(\tau \right)\;d\tau$, the system can be rewritten as a second-order state-space representation:

(37) $$\begin{array}{cc} \dot{e}\left(t\right) & =\frac{k_{1}-f_{v}}{J}e\left(t\right)+\frac{k_{2}}{J}\xi \left(t\right), \end{array}$$

(38) $$\begin{array}{cc} \dot{\xi }\left(t\right) & =e(t). \end{array}$$

Applying the Laplace transform yields

(39) $$Js^{2}E\left(s\right)=\left(k_{1}-f_{v}\right)sE\left(s\right)+k_{2}E\left(s\right),$$

which leads to the characteristic equation

(40) $$s^{2}+\frac{f_{v}-k_{1}}{J}s-\frac{k_{2}}{J}=0.$$

For a second-order system described by the standard polynomial $s^{2}+a_{1}s+a_{0}=0$, the Routh–Hurwitz stability criterion requires that all coefficients be positive. From (40), the following conditions are obtained:

(41) $$a_{1}=\frac{f_{v}-k_{1}}{J}>0\;\Rightarrow \;k_{1}<f_{v},$$

(42) $$a_{0}=-\frac{k_{2}}{J}>0\;\Rightarrow \;k_{2}<0.$$

These inequalities are consistent with the stability conditions stated after (34).

##### Gain Selection Methodology

*Step 1: Specification of desired closed-loop dynamics.* The target closed-loop performance specifications were chosen as follows:

- Settling time: $t_{s}\approx 2\;s$, typical for servo motor applications;
- Damping ratio: ζ≈0.7, allowing slight overshoot while maintaining good transient response.

*Step 2: Relationship between specifications and characteristic equation.* For a standard second-order system described by

(43) $$s^{2}+2\zeta \omega_{n}s+\omega_{n}^{2}=0,$$

the natural frequency $\omega_{n}$ is related to the settling time (2% criterion) by

(44) $$t_{s}=\frac{4}{\zeta \omega_{n}}.$$

Substituting $t_{s}=2\;s$ and ζ=0.7 yields

(45) $$\omega_{n}=\frac{4}{\zeta t_{s}}=\frac{4}{0.7\times 2}=2.857\;\mathrm{rad}/s.$$

*Step 3: Matching characteristic equation coefficients.* Comparing (40) with the standard form results in

(46) $$\begin{array}{cc} 2\zeta \omega_{n}= & \frac{f_{v}-k_{1}}{J}\;\Rightarrow \;k_{1}=f_{v}-J\left(2\zeta \omega_{n}\right), \end{array}$$

(47) $$\begin{array}{cc} \omega_{n}^{2}= & -\frac{k_{2}}{J}\;\Rightarrow \;k_{2}=-J\omega_{n}^{2}. \end{array}$$

Substituting the identified parameters $J=0.1346\;\mathrm{kg}\cdot m^{2}$ and $f_{v}=0.3935\;N\cdot m\cdot s$ gives

(48) $$\begin{array}{cc} k_{1} & =-0.144\;N\cdot m\cdot s, \end{array}$$

(49) $$\begin{array}{cc} k_{2} & =-0.110\;N\cdot m. \end{array}$$

*Step 4: Practical adjustment and validation.* The theoretically obtained gains $(k_{1}=-0.144,$ $k_{2}=-0.110)$ were slightly adjusted to $\left(k_{1}=-0.2,\;k_{2}=-0.1\right)$ based on practical considerations:

- Increasing the magnitude of $k_{1}$ improves disturbance rejection at the expense of a moderate increase in overshoot;
- Reducing the magnitude of $k_{2}$ mitigates integral windup effects at low velocities.

**Figure 17:**
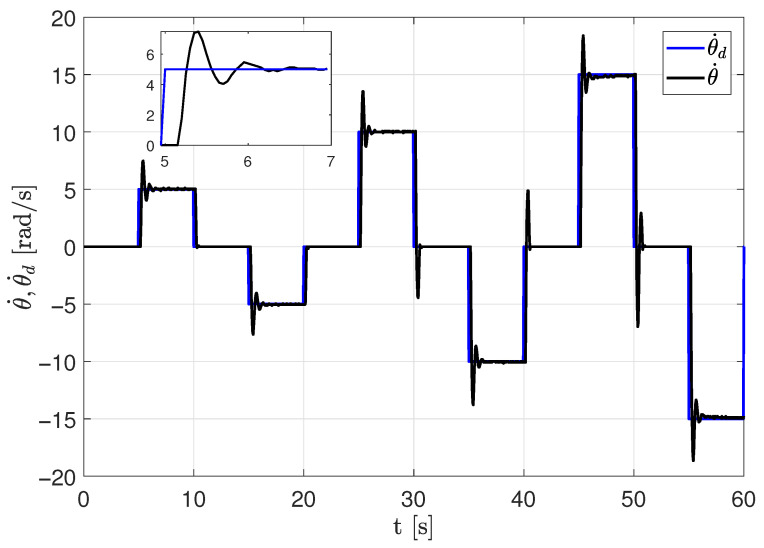
Performance of velocity using the identified parameters and PI closed-loop control.

**Figure 18:**
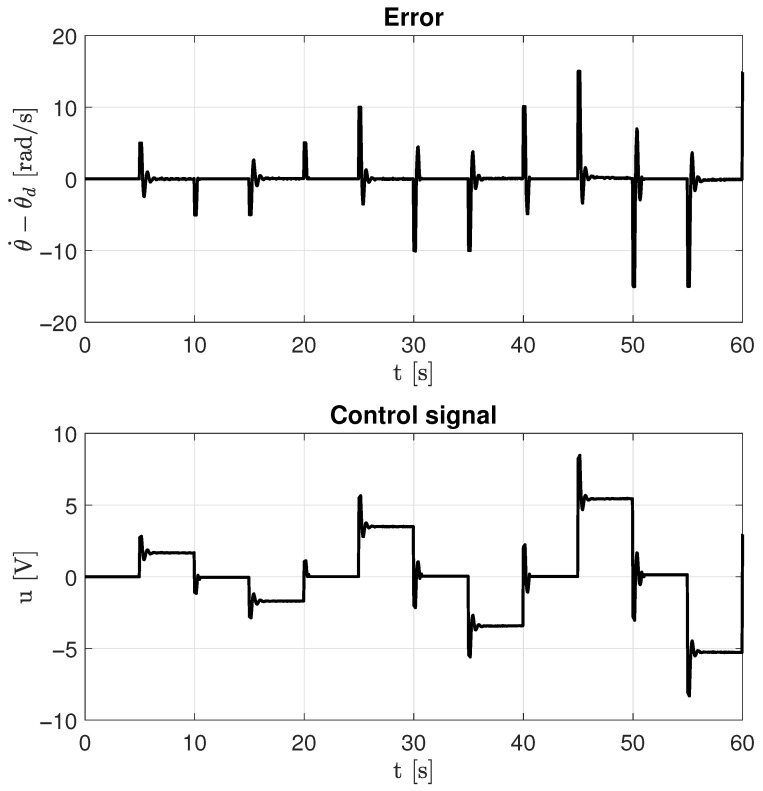
Error and control input performance using the identified parameters and PI closed-loop control.

The experimental results presented in Figure 17 and Figure 18 demonstrate precise velocity tracking with minimal steady-state error. The system maintains stability even when the reference velocity exceeds the identified linear region, confirming that the identified model parameters accurately describe the system dynamics and can be effectively used for control design.

## 5. Conclusions and Discussion

The experimental results presented in this work demonstrate that both the constant torque and ramp-type excitation methods provide consistent and reliable estimates of the viscous friction, Coulomb friction, and inertia coefficients of a DC motor. The viscous friction coefficients obtained by both methods are nearly identical, confirming the linear dependence of this component on angular velocity. This consistency directly supports the accuracy of the proposed analytical models and validates their applicability for controller design.

The Coulomb friction coefficient, on the other hand, showed lower values when using the ramp-type input method. This can be attributed to the continuous acceleration inherent to this excitation, which reduces the influence of static friction compared to the constant torque case, where acceleration is zero. Although Coulomb friction has a smaller impact on high-speed control, it remains critical at low velocities where nonlinear effects dominate. The inertia coefficient values estimated by both methods showed close agreement, demonstrating the robustness of the analytical expressions derived for transient response analysis.

The identified parameters were used in the implementation of a proportional–integral (PI) velocity controller, achieving stable and accurate tracking performance. The control system maintained robustness within and slightly beyond the linear operating region, validating the fidelity of the identified parameters in dynamic control applications. These results confirm that direct analytical estimation methods, free of iterative or optimization-based computations, can yield precise and computationally efficient parameter identification results, suitable for educational and embedded applications with limited processing resources.

### Discussion and Limitations

Despite the overall effectiveness of the proposed identification framework, several limitations must be acknowledged. First, the experimental setup was restricted to a single DC motor model operating under laboratory conditions and without variable load disturbances. Consequently, the identified parameters can vary under different environmental conditions, such as changes in temperature or humidity, which can alter frictional behavior and material properties. In addition, nonlinear friction effects such as Stribeck behavior and stick–slip dynamics were not explicitly modeled, which may limit the accuracy of the identified parameters at very low velocities.

Another limitation is that this study focused on open-loop identification with relatively simple excitation profiles. More complex system identification techniques, such as frequency-domain analysis or observer-based estimation, could improve the robustness of the results against measurement noise or unmodeled dynamics.

## Figures and Tables

**Table 1:** Summary of friction models, identification parameters, and experimental requirements.

Friction Model	Model Type	Parameters to Be Identified	Required Experimental Conditions
Viscous friction	Static, linear	$f_{v}$	Steady-state tests at constant velocity
Coulomb + viscous friction	Static, nonlinear	$f_{c},\;f_{v}$	Steady-state tests with positive and negative velocities
Coulomb + viscous with bias	Static, nonlinear	$f_{c},\;f_{v},\;b$	Steady-state tests under unidirectional rotation
Static LuGre (Stribeck effect)	Static, nonlinear	$f_{c},\;f_{s},\;f_{v},\;\omega_{s}$	Low-speed velocity sweeps in both directions
Dahl model	Dynamic, nonlinear	$\sigma_{0},\;f_{c}$	Torque or voltage step and ramp tests
Dynamic LuGre model	Dynamic, nonlinear	$\sigma_{0},\;\sigma_{1},\;f_{c},\;f_{s},\;f_{v},\;\omega_{s}$	Step, ramp, or PRBS excitations with persistent excitation
Dynamic LuGre with inertia	Dynamic, nonlinear	$J,\;\sigma_{0},\;\sigma_{1},\;f_{c},\;f_{s},\;f_{v},\;\omega_{s}$	PRBS or multi-step tests with velocity reversals
Pure mechanical dynamic model	Dynamic, linear	J,b	Step response tests neglecting nonlinear friction effects

**Table 2:** Viscous and Coulomb friction coefficients derived using the constant torque method.

Input Voltage	$f_{v}$	$|f_{c}|$
Positive (0 to 12 V)	0.3945	0.4694
Positive (12 to 0 V)	0.3999	0.5954
Negative (0 to −12 V)	0.3869	0.4334
Negative (−12 to 0 V)	0.3929	0.5586
Average Value	0.3935	0.5141

Equation of the straight line: $u=f_{v}\dot{\theta }+f_{c}$, where *m* is the slope and *c* is the y-intercept.

**Table 3:** Viscous and Coulomb friction coefficients derived using the ramp-type input method.

Parameter	Positive Ramp	Negative Ramp
*r*	0.2 V/s	−0.2 V/s
*m*	0.5002	−0.5230
*b*	1.2205	−0.8149
$f_{v}$	0.3998 N·m·s	0.3824 N·m·s
$f_{c}$	0.4880 N·m	0.3116 N·m
*r*	0.5 V/s	−0.5 V/s
*m*	1.2330	−1.3041
*b*	1.1143	−0.3868
$f_{v}$	0.4055 N·m·s	0.3834 N·m·s
$f_{c}$	0.4519 N·m	0.1483 N·m
*r*	0.94 V/s	−0.94 V/s
*m*	2.2978	−2.4029
*b*	0.7809	−0.6552
$f_{v}$	0.4091 N·m·s	0.3912 N·m·s
$f_{c}$	0.3195 N·m	0.2563 N·m

r= ramp slope, m= velocity slope, b= intercept with the velocity axis, $f_{v}=$ viscous friction coefficient and $f_{c}=$ Coulomb friction coefficient.

**Table 4:** Viscous and Coulomb friction coefficients estimated by both methods.

Parameter	Constant Torque	Ramp
Viscous Friction	0.3935 N·m·s	0.3952 N·m·s
Coulomb Friction	0.5141 N·m	0.3293 N·m

**Table 5:** Comparison of friction models using steady-state experimental data.

Model	Parameters	RMSE (N·m)	$R^{2}$	Func. Eval.
Coulomb–viscous	$f_{c},\;f_{v}$	0.29	0.982	34
Stribeck	$f_{c},\;f_{s},\;f_{v},\;\omega_{s}$	0.18	0.991	97
LuGre (steady-state)	$\sigma_{0},\;\sigma_{1},\;f_{v},\;f_{s}$	0.20	0.990	112

**Table 6:** Estimated moment of inertia using parameters from both methods.

Parameter	Constant Torque	Ramp
Viscous Friction	0.3935 N·m·s	0.3952 N·m·s
Coulomb Friction	0.5141 N·m	0.3293 N·m
Moment of Inertia	0.1346 kg·m^2^	0.1178 kg·m^2^

**Table 7:** Repeatability Statistics for Constant Torque Method (5 trials per condition).

Input Voltage Direction	Viscous Friction $f_{v}$			Coulomb Friction $f_{c}$		
	Mean (N·m·s)	Std Dev (N·m·s)	RSD (%)	Mean (N·m)	Std Dev (N·m)	RSD (%)
Positive (0 to 12 V)	0.3945	0.0108	2.74	0.4694	0.0187	3.98
Positive (12 to 0 V)	0.3999	0.0121	3.03	0.5954	0.0245	4.12
Negative (0 to −12 V)	0.3869	0.0095	2.45	0.4334	0.0163	3.76
Negative (−12 to 0 V)	0.3929	0.0114	2.90	0.5586	0.0219	3.92
**Overall Average**	**0.3935**	**0.0110**	**2.78**	**0.5141**	**0.0204**	**3.95**

RSD = Relative Standard Deviation = (σ/μ)×100%.

**Table 8:** Repeatability Statistics for Ramp Input Method (5 trials per condition).

Ramp Rate	Viscous Friction $f_{v}$			Coulomb Friction $f_{c}$		
	Mean (N·m·s)	Std Dev (N·m·s)	RSD (%)	Mean (N·m)	Std Dev (N·m)	RSD (%)
+0.2 V/s	0.3998	0.0127	3.18	0.4880	0.0312	6.39
+0.5 V/s	0.4055	0.0133	3.28	0.4519	0.0289	6.40
+0.94 V/s	0.4091	0.0142	3.47	0.3195	0.0208	6.51
−0.2 V/s	0.3824	0.0115	3.01	0.3116	0.0201	6.45
−0.5 V/s	0.3834	0.0119	3.10	0.1483	0.0098	6.61
−0.94 V/s	0.3912	0.0128	3.27	0.2563	0.0167	6.52
**Overall Average**	**0.3952**	**0.0127**	**3.22**	**0.3293**	**0.0213**	**6.48**

**Table 9:** Repeatability Statistics for Moment of Inertia (8 trials).

Method	Mean *J* (kg·m^2^)	Std Dev *J* (kg·m^2^)	RSD *J* (%)	95% Confidence Interval (kg·m^2^)
Using constant torque friction parameters	0.1346	0.0041	3.05	[0.1313, 0.1379]
Using ramp input friction parameters	0.1178	0.0048	4.08	[0.1140, 0.1216]

**Table 10:** Comprehensive Statistical Summary of Parameter Identification Repeatability.

Parameter	Method	Trials (*n*)	Mean	Std Dev	RSD (%)	95% CI	Range (Min–Max)
Viscous Friction $f_{v}$ (N·m·s)	Constant Torque	20	0.3935	0.0110	2.78	[0.3830, 0.4040]	[0.3781, 0.4103]
	Ramp Input	30	0.3952	0.0127	3.22	[0.3845, 0.4059]	[0.3765, 0.4145]
Coulomb Friction $f_{c}$ (N·m)	Constant Torque	20	0.5141	0.0204	3.95	[0.5049, 0.5233]	[0.4823, 0.5512]
	Ramp Input	30	0.3293	0.0213	6.48	[0.3196, 0.3390]	[0.3012, 0.3621]
Moment of Inertia *J* (kg·m^2^)	Transient (CT friction)	8	0.1346	0.0041	3.05	[0.1313, 0.1379]	[0.1289, 0.1398]
	Transient (Ramp friction)	8	0.1178	0.0048	4.08	[0.1140, 0.1216]	[0.1117, 0.1243]

CT = Constant Torque method; RSD = Relative Standard Deviation = (Std Dev/Mean) × 100%. 95% CI = 95% Confidence Interval assuming normal distribution: [μ−1.96σ, μ+1.96σ].

## Data Availability

The original contributions presented in this study are included in the article. Further inquiries can be directed to the corresponding author.

## References

[1] Kim S. (2018). Moment of inertia and friction torque coefficient identification in a servo drive system. IEEE Trans. Ind. Electron..

[2] Freidovich L., Robertsson A., Shiriaev A., Johansson R. (2009). LuGre-model-based friction compensation. IEEE Trans. Control Syst. Technol..

[3] Zimmermann K., Zeidis I., Lysenko V. (2021). Mathematical model of a linear motor controlled by a periodic magnetic field considering dry and viscous friction. Appl. Math. Model..

[4] Kim N.J., Moon H.S., Hyun D.S. (1996). Inertia identification for the speed observer of the low speed control of induction machines. IEEE Trans. Ind. Appl..

[5] Lee K.B., Blaabjerg F. (2007). Robust and stable disturbance observer of servo system for low-speed operation. IEEE Trans. Ind. Appl..

[6] Seong H., Chung C., Shim D. (2023). Model Parameter Identification via a Hyperparameter Optimization Scheme for Autonomous Racing Systems. IEEE Control Syst. Lett..

[7] Batool A., ul Ain N., Amin A.A., Adnan M., Shahbaz M.H. (2022). A comparative study of DC servo motor parameter estimation using various techniques. Automatika.

[8] Fazdi M.F., Hsueh P.W. (2023). Parameters Identification of a Permanent Magnet DC Motor: A Review. Electronics.

[9] Kuczmann M. (2024). Review of DC Motor Modeling and Linear Control: Theory with Laboratory Tests. Electronics.

[10] Wu W. (2012). DC Motor Parameter Identification Using Speed Step Responses. Model. Simul. Eng..

[11] Brablc M., Sova V., Grepl R. Adaptive feedforward controller for a DC motor drive based on inverse dynamic model with recursive least squares parameter estimation. Proceedings of the 2016 17th International Conference on Mechatronics—Mechatronika (ME).

[12] De Souza D.A., Batista J.G., Vasconcelos F.J.S., Dos Reis L.L.N., Machado G.F., Costa J.R., Junior J.N.N., Silva J.L.N., Rios C.S.N., Júnior A.B.S. (2021). Identification by Recursive Least Squares with Kalman Filter (RLS-KF) Applied to a Robotic Manipulator. IEEE Access.

[13] Chebbi A., Franchek M.A., Grigoriadis K. (2025). Simultaneous State and Parameter Estimation Methods Based on Kalman Filters and Luenberger Observers: A Tutorial and Review. Sensors.

[14] Brosch A., Hanke S., Wallscheid O., Böcker J. (2021). Data-Driven Recursive Least Squares Estimation for Model Predictive Current Control of Permanent Magnet Synchronous Motors. IEEE Trans. Power Electron..

[15] Amiri M.S., Ibrahim M.F., Ramli R.B. (2020). Optimal parameter estimation for a DC motor using genetic algorithm. Int. J. Power Electron. Drive Syst..

[16] Rodríguez-Abreo O., Hernandez-Paredes J.M., Rangel A.F., Fuentes-Silva C., Velásquez F.A.C. (2021). Parameter Identification of Motors by Cuckoo Search Using Steady-State Relations. IEEE Access.

[17] Karnavas Y.L. (2020). Application of recent nature-inspired meta-heuristic optimisation techniques to small permanent magnet DC motor parameters identification problem. J. Eng..

[18] Gökçe C.O., İpek M.E., Dayıoğlu M., Ünal R. (2025). Parameter estimation and speed control of real DC motor with low resolution encoder. Results Control Optim..

[19] Siddiqi F.U.R., Ahmad S., Akram T., Ali M.U., Zafar A., Lee S.W. (2024). Artificial Neural Network-Based Data-Driven Parameter Estimation Approach: Applications in PMDC Motors. Mathematics.

[20] Olejnik P., Ayankoso S. (2023). Friction modelling and the use of a physics-informed neural network for estimating frictional torque characteristics. Meccanica.

[21] Balara D., Timko J., Žilková J., Lešo M. (2017). Neural networks application for mechanical parameters identification of asynchronous motor. Neural Netw. World.

[22] Virgala I., Kelemen M. (2013). Experimental friction identification of a DC motor. Int. J. Mech. Appl..

[23] Traversaro S., Prete A.D., Muradore R., Natale L., Nori F. Inertial parameter identification including friction and motor dynamics. Proceedings of the 13th IEEE-RAS International Conference on Humanoid Robots.

[24] Kelly R., Llamas J., Campa R. (2000). A measurement procedure for viscous and coulomb friction. IEEE Trans. Instrum. Meas..

[25] Ponce I., Orlov Y., Cuesta Garcia J. Comparacion de dos metodos de estimacion de los parametros de las fricciones viscosa y de Coulomb para un motor de CD. Proceedings of the Congreso de Instrumentacion SOMI XXX.

[26] Yerlikaya U., Balkan T. Identification of Viscous and Coulomb Friction in Motion Constrained Systems. Proceedings of the 2018 IEEE/ASME International Conference on Advanced Intelligent Mechatronics (AIM).

[27] De Wit C.C., Olsson H., Astrom K.J., Lischinsky P. (1995). A new model for control of systems with friction. IEEE Trans. Autom. Control.

[28] Soto I., Campa R., Sánchez-Mazuca S. (2020). Modelado y control con compensacion de friccion de un sistema pendubot. Revista Iberoamericana de Automática e Informática Industrial.

[29] Olsson H. (1996). Control Systems with Friction. Doctoral Thesis.

[30] Piatkowski T. (2014). Dahl and LuGre dynamic friction models—The analysis of selected properties. Mech. Mach. Theory.

[31] Hernandez E.D.R., Garcia B.E.S., Cortes F.R., Trevino M.A.V., Barriga J.L.O. Nuevo modelo de friccion para robots manipuladores. Proceedings of the Mem. ELECTRO.

[32] Yao J., Jiao Z., Ma D. (2014). RISE-Based Precision Motion Control of DC Motors With Continuous Friction Compensation. IEEE Trans. Ind. Electron..

[33] Bhushan B. (2013). Introduction to Tribology.

[34] Olsson H., Åström K., Canudas de Wit C., Gäfvert M., Lischinsky P. (1998). Friction Models and Friction Compensation. Eur. J. Control.

[35] Virgala I., Frankovsky P., Kenderova M. (2013). Friction Effect Analysis of a DC Motor. Am. J. Mech. Eng..

[36] Armstrong-Helouvry B., Dupont P., De Wit C.C. (1994). A survey of models, analysis tools and compensation methods for the control of machines with friction. Automatica.

[37] Kaewkham-ai B., Uthaichana K. Comparative study on friction compensation using Coulomb and Dahl models with extended and unscented Kalman filters. Proceedings of the 2012 7th IEEE Conference on Industrial Electronics and Applications (ICIEA).

[38] Xu Z.D., Guo Y.Q., Zhu J.T., Xu F.H., Xu Z.D., Guo Y.Q., Zhu J.T., Xu F.H. (2017). Chapter 4—Semiactive Intelligent Control. Intelligent Vibration Control in Civil Engineering Structures.

